# The American Protologic Society Prize for the Best Original Essay on Any Disease of the Colon

**Published:** 1911-02

**Authors:** 


### Abstracts and Selections.

### The American Protologic Society Prize for the Best Original Essay on Any Disease of the Colon.

The American Protologic Society announces through its committee that the cash sum of $100 will be awarded, as soon as possible in 1911, to the author of the best original essay on any disease of the colon in competition for the above prize.

Essays must be submitted, to the secretary of the committee, on or before May 10, 1911. The address of the secretary is given below, to whom all communications should be addressed.

Each essay must be typewritten, *designated by a motto or device, and without signature or any other indication of its authorship, and be accompanied by a separate sealed envelope, having on its outside only the motto or device contained on the essay, and within the name, the motto or device used on the essay, and, the address of the author.* No envelope will be opened except that which accompanies the successful essay.

The committee will return the unsuccessful essays, if reclaimed by their writers within six months, provided return postage accompanies the application.

The committee reserves the right not to make an award if no essay submitted is considered worthy of the prize.

The competition is open to graduates of medicine (not fellows of the Society) and to members of the senior classes of all colleges in the United States or Canada.

The object of the prize and competition is to stimulate an increased interest in, and knowledge of, proctology.

The committee shall have full control of awarding the prize and the publication of the prize essay, and it shall be the property of the American Protologic Society. It may be published in the Transactions of the Society and also as a separate issue if deemed expedient. The committee may increase its membership if deemed advisable.

DR. DWIGHT H. MURRAY, Chairman;
DR. SAMUEL T. EARLE,
DR. JEROME M. LYNCH,
DR. ALOIS B. GRAHAM,
DR. LEWIS H. ADLER, JR., Secretary;

1610 Arch St., Philadelphia, Pa. Committee.

